# Impact of body weight and sex in selected dog breeds on the canine adrenal gland dimensions measured by computed tomographic imaging

**DOI:** 10.1186/s12917-023-03641-0

**Published:** 2023-07-29

**Authors:** Gerrit Büttelmann, Lisa Katharina Harder, Ingo Nolte, Patrick Wefstaedt

**Affiliations:** Small Animal Clinic, University of Veterinary Medicine Hannover, Foundation, Bünteweg 9, D- 30559 Hannover, Germany

**Keywords:** Adrenal gland, Attenuation, Amira, Body weight, Breeds, Computed tomography, Dogs, Quantification, Volume

## Abstract

**Background:**

The present study aimed to investigate possible influences of body weight and sex on adrenal gland size in endocrinologically healthy dogs. Possible factors influencing the adrenal size are discussed in relation to a universal upper reference value from the literature of 7.4 mm as the thickness in the caudal pole of the adrenal gland. The adrenal size was measured by computed tomography (CT) from 66 normal dogs of six different breeds (Labrador Retriever (n = 16), German Shepherd (n = 10), Boxer (n = 8), Beagle (n = 14), Dachshund (n = 6) and Jack Russell Terrier (n = 12); male n = 38 (thereof neutered n = 23), female n = 28 (thereof neutered n = 17)) based on volume quantification and linear measurements using the data processing software Amira. For interbreed comparability, a ratio consisting of the third root of adrenal volume to aortic diameter (Ratio volume-aorta, RVA) was introduced. Additionally, breed-related attenuation values in contrast-enhanced CT data sets were measured.

**Results:**

The measured volumes ranged from 0.34 to 1.93 cm^3^ for the right and from 0.39 to 2.23 cm^3^ for the left adrenal gland. The present study was able to demonstrate a body weight effect on the adrenal volume as well as on length and height. In terms of adrenal size, no significant differences between male and female, nor between intact and neutered dogs were obtained due to the RVA. In addition, for the weight classes, a breed independent threshold for dogs less (left 1.4; right gland 1.5) or more than 20 kg body weight (left 1.1; right gland 1.2) based on RVA was defined. Breed-related significant differences with respect to attenuation were determined only for the left adrenal gland, with lower attenuation values in large dog breeds.

**Conclusion:**

The present study points out the importance of weight-related data when assessing CT data of the canine adrenal gland regarding volume, size and attenuation. The use of a universal reference value for the assessment of adrenal size appears unsuitable considering weight-related volume and linear measurements. Sex seems not to affect adrenal gland size.

## Background

Diseases of the adrenal gland are common in dogs and most often associated with enlargement of one or both adrenal glands [1]. Apart from endocrine screening tests, gland size has been used as the principal imaging criterion to differentiate an unaltered adrenal gland from adrenal pathologies [2, 3]. Computed tomography (CT) is recommended in human medicine as a beneficial tool for assessing the adrenal size, morphology and possible vascular invasion in presence of neoplasia [4–7]. As a noninvasive imaging technique secondary to endocrine testing, ultrasonography is the preferred method for assessing adrenal morphology in small animals [3, 8, 9]. Due to the rising availability of CT in veterinary medicine, the opportunity for clinical evaluation of the adrenal glands has increased. Previous studies reported CT findings and cross-sectional measurements of normal canine adrenal glands [10], but also of canine hyperadrenocorticism [11, 12] and adrenal neoplasia [13]. Linear measurements were primarily used to characterize adrenal size [10–13], whereas the application of volumetric measurements was not considered. The use of volume measurements from CT scans has been described as an accurate method to assess the size of other abdominal organs [14, 15]. However, in one CT imaging study, the use of simple cross-sectional measurements compared with volume quantification of the human adrenal gland was judged to have reduced reproducibility due to a low interobserver reliability [16]. Since focal nodular alterations (as described in adenomas, metastases, and hyperplasia) may occur instead of nonspecific adrenal enlargement [17], volume quantification appears to be a more appropriate tool for describing adrenal size by use of numerous sampling points compared to focal linear measurements. So far there are only a few studies that have examined the volume of canine adrenal glands using computed tomography. Bertolini et al. (2006, 2008) were able to show that ACTH-dependent hyperadrenocorticism in dogs is associated with increased adrenal gland volume [8, 18]. Thereby, volumes of unaltered adrenal glands were quantified for the control group mainly in endocrinologically healthy dogs of small to medium body weight [8, 18]. Thus, no evidence concerning possible effects of body weight on adrenal volume is currently available. In this context, ultrasonographic studies using linear length and cross-sectional measurements indicate a positive correlation between body weight and adrenal size with respect to adrenal length and height [9, 19, 20]. However, an upper threshold of 7.4 mm of maximal diameter in the caudal adrenal pole, independent of body weight or breed for the diagnosis of ACTH-dependent hyperadrenocorticism, has been discussed in the literature [21]. In the context of possible weight-dependent adrenal size, the utility of a weight-independent reference value needs to be reviewed. Therefore, the present study was performed to evaluate the hypothesis that adrenal size is influenced by body weight using volume quantification and simple linear measurements. Additionally, to the best of our knowledge, no sex-related data regarding adrenal volume of unaltered glands have been collected in the literature. For this reason, the hypothesis of possible sex differences was also intended to be tested in selected breeds.

In addition to volume measurements, evaluation of attenuation values on native and postcontrast CT images provides information on underlying tissue characteristics and the possibility of diagnosing adrenal gland neoplasia [13, 22]. However, only limited data are available for attenuation values of unaltered adrenal glands from endocrinologically healthy dogs that do not involve the entire organ and include larger contrast enhanced vessels [8]. Thereby, the inclusion of the contrast agent-carrying vascular structures may lead to increased attenuation values. In contrast to the study of Bertolini et al. (2006), adrenal attenuation values in enhanced CT scans were determined using a three-dimensional region of interest (ROI) excluding all attached vascular structures, in particular the phrenicoabdominal vein and artery. Thus, the aim of the present study was to establish further information on possible breed-related differences of adrenal attenuation values at standardized acquisition protocol excluding vascular patterns using a spatial measurement method.

## Methods

### Subjects

In this retrospective study, CT data sets of dogs that had been examined previously at the Small Animal Clinic, University of Veterinary Medicine Hannover, Foundation, Germany due to other diseases unrelated to the adrenal glands were reviewed. Thus, dogs aged at least two years that did not show evidence of endocrine disease (e.g., hyperadrenocorticism, hypothyroidism) based on the patient’s history, including a complete physical examination and the results of hematologic and serum biochemical analyses performed maximum two days prior or on the day of the CT scans, were enrolled in the study. Patients receiving glucocorticoid treatment within 12 months prior to this CT examination were excluded. From the database, six different breeds representing various weight classes were selected for the present study. The selected dog breeds included the Labrador Retriever (n = 16), German Shepherd (n = 10), Boxer (n = 8), Beagle (n = 14), Dachshund (n = 6) and Jack Russell Terrier (n = 12). Information on sex, body weight and age in the respective breed groups is listed in the following table (Table 1).

**Table 1:** Breed related information of included sample size, castration status, age and body weight

	Male intact (N)	Female intact (N)	Male neutered (N)	Female neutered (N)	Total number (N)	Age (Years)	Body weight (kg)
German Shepherd	1	1	3	5	10	8.2 ± 3.1	34.2 ± 8.4
Labrador Retriever	3	4	5	4	16	7.2 ± 3.2	34.2 ± 6.6
Boxer	3	1	3	1	8	8.8 ± 2.1	36.4 ± 4.3
Beagle	2	1	7	4	14	7.3 ± 4.3	14.2 ± 3.9
Jack Russel Terrier	1	3	5	3	12	10.5 ± 3.7	7.9 ± 2.4
Dachshund	5	1	0	0	6	9.8 ± 2.6	8.4 ± 2.6
Total number (N)	15	11	23	17			

The present table lists the sample numbers (N) of the selected dog breeds included in this study, considering the neutering status as well as mean age and body weight (± SD)

### CT data acquisition

Each CT examination data set was acquired with a 64-multi-detector-row CT scanner (Phillips Brilliance 64, Philips GmbH, Hamburg, Germany). The abdominal CT scans performed in dorsal or ventral recumbency were obtained with a voltage of 120 kV, slice thickness of 2 mm, pitch factor of 1.171 and a pixel size resolution ranging from 0.26 × 0.26 mm to 0.97 × 0.97 mm. Additionally, the pixel size was interpolated to 0.2 × 0.2 mm to achieve a uniform resolution between CT scans. To reduce the radiation dose for the patient, an automatic current selection function (DoseRight-D) was used as standard during tube rotation, which adjusts the amperage depending on the changes in body symmetry. As a result, different mAs-products were generated for each individual CT scan. For the examination, patients were anesthetized with levomethadon (L-Polamivet® 0.2 mg/kg, MSD Tiergesundheit/Intervet Deutschland GmbH, Unterschleißheim, Germany), diazepam (Ziapam®, 0.5 mg/kg, Laboratoire TVM, Lempdes, France), and propofol (individual dose depending on effect; Narcofol®, CP-Pharma Handelsgesellschaft mbH, Burgdorf, Germany). During the CT examination, anesthesia was maintained by inhalation with isoflurane (Isofluran CP®, CP-Pharma Handelsgesellschaft mbH). Following the performance of a native scan, a second scan was carried out after administering a non-ionic iodinated contrast agent (Xenetix® 300, Guerbet GmbH; Sulzbach, Germany) by injection into the vena saphena or the vena cephalica antebrachii using a power injector (MedRad Vistron CT® 610 System, MedRad, Inc., Indianola, PA, USA, dosage: 2 mL/kg body weight; flow rate: max. 3 mL/sec; duration: max. 30 s). For the automatical start of the second scan a local ROI was placed at the level of the diaphragm within the aorta in transversal image view. The abdominal CT scan was initiated 49 s after a threshold of 150 Hounsfield Units (HU) had been attained within this ROI. All CT data sets were stored in DICOM format. To exclude adrenal endocrine inactive neoplasms or cystic alterations as a source of error, exclusively adrenal glands with homogeneous contrast agent distribution were regarded. Furthermore, only contrast-enhanced abdominal CT examination data sets with both adrenal glands completely assessable were used. The morphological evaluation of the adrenal glands was performed at an image-processing workstation (Extended Brilliance Workspace, Philips Medical System, Inc., Cleveland, OH, USA).

### Evaluation of adrenal volume and attenuation

**Fig. 1:**
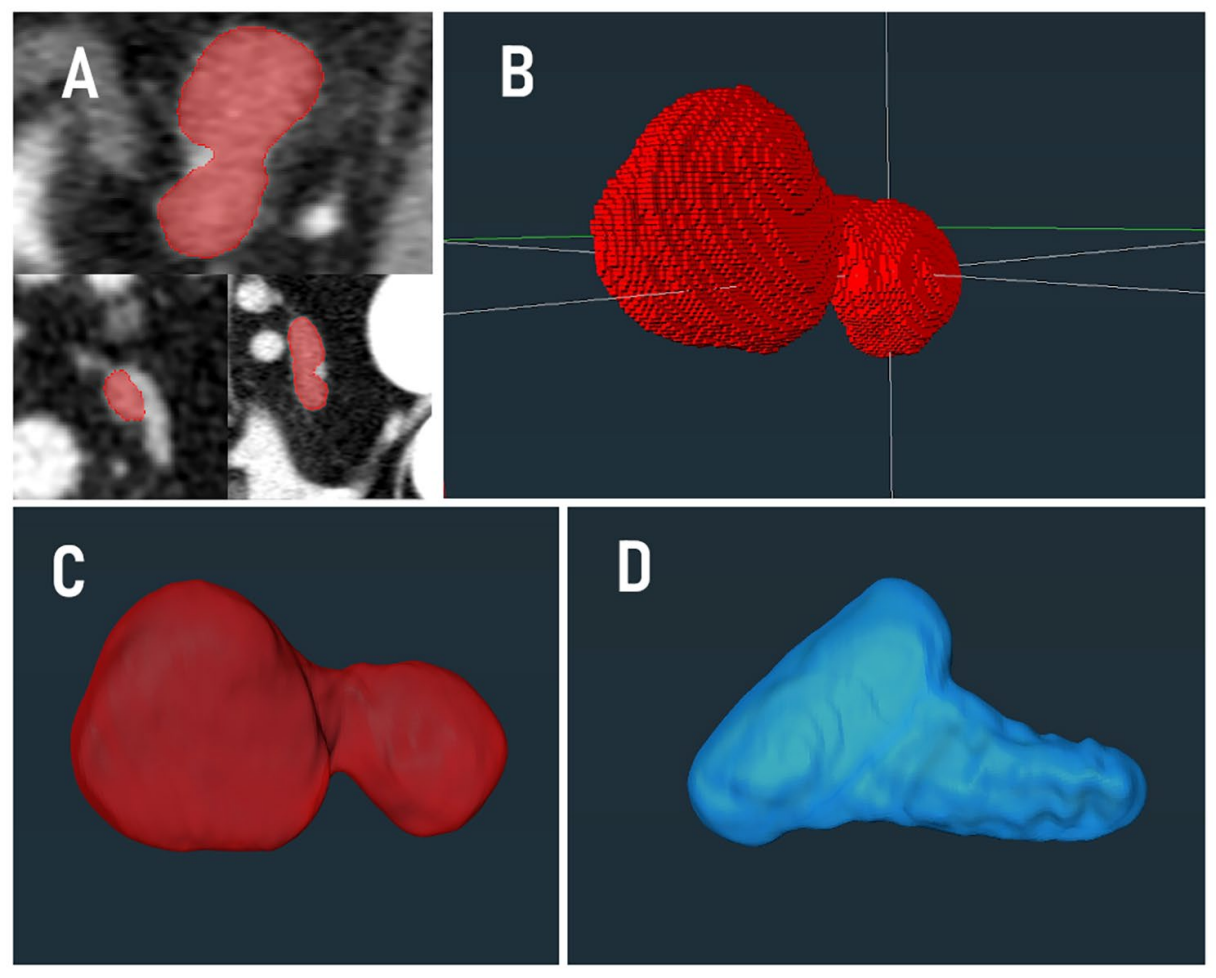
Slice addition technique. Quantification of the canine adrenal gland using the Amira 6.5 software program applying a slice addition technique (SAT). Figure 1.A: For volume and attenuation measurements, the sectional area of each adrenal gland was manually marked slice by slice in the sagittal image plane. During segmentation, vena and arteria phrenicoabdominalis were excluded from the measurements under visual control in the transversal and dorsal projection planes. Figure 1.B: Voxels assembled from the previous selection to form a three-dimensional illustration of the canine adrenal gland. Figure 1.C: Three-dimensional surface reconstruction of a left adrenal gland. Figure 1.D: Three-dimensional surface of a right adrenal gland created using the same imaging technique

The adrenal gland volume was measured using a software for three-dimensional data visualization, processing, and analysis (Amira 6.5, FEI, part of Thermo Fischer Scientific, Inc., Hillsboro, OR, USA). Each adrenal gland was measured by the same observer. For volume quantification, adrenal segmentation of the target organ from surrounding tissue was required. Automatic segmentation procedures could not be applied due to the low contrast difference between the adrenal glands and the surrounding structures. Thus, manual segmentation was performed in contrast -enhanced CT scans for volume determination. Accordingly, the organ borders of the adrenal gland were encircled within each slice manually with the mouse cursor in sagittal image view. The adrenal-associated phrenicoabdominal vein and artery were excluded from the volumetric measurements. In order to determine the adrenal volume, for each selected slice, the corresponding number of voxels in the marked area multiplied by the size of a single voxel was added. The partial volumes calculated for each slice were added up to obtain the total volume of the adrenal gland. From the voxels added in the slice addition technique (SAT) procedure, a three-dimensional reconstruction of the adrenal surface was computed and displayed by the software Amira 6.5 (Fig. 1).

Adrenal attenuation was measured in Hounsfield Units (HU). An average attenuation value was calculated based on all voxels selected for volumetric measurements of the respective adrenal gland in contrast-enhanced CT scan. Thus, the chosen three-dimensional ROI referred to the entire endocrine organ, including the boundary areas.

### Length measurements of the adrenal glands

**Fig. 2:**
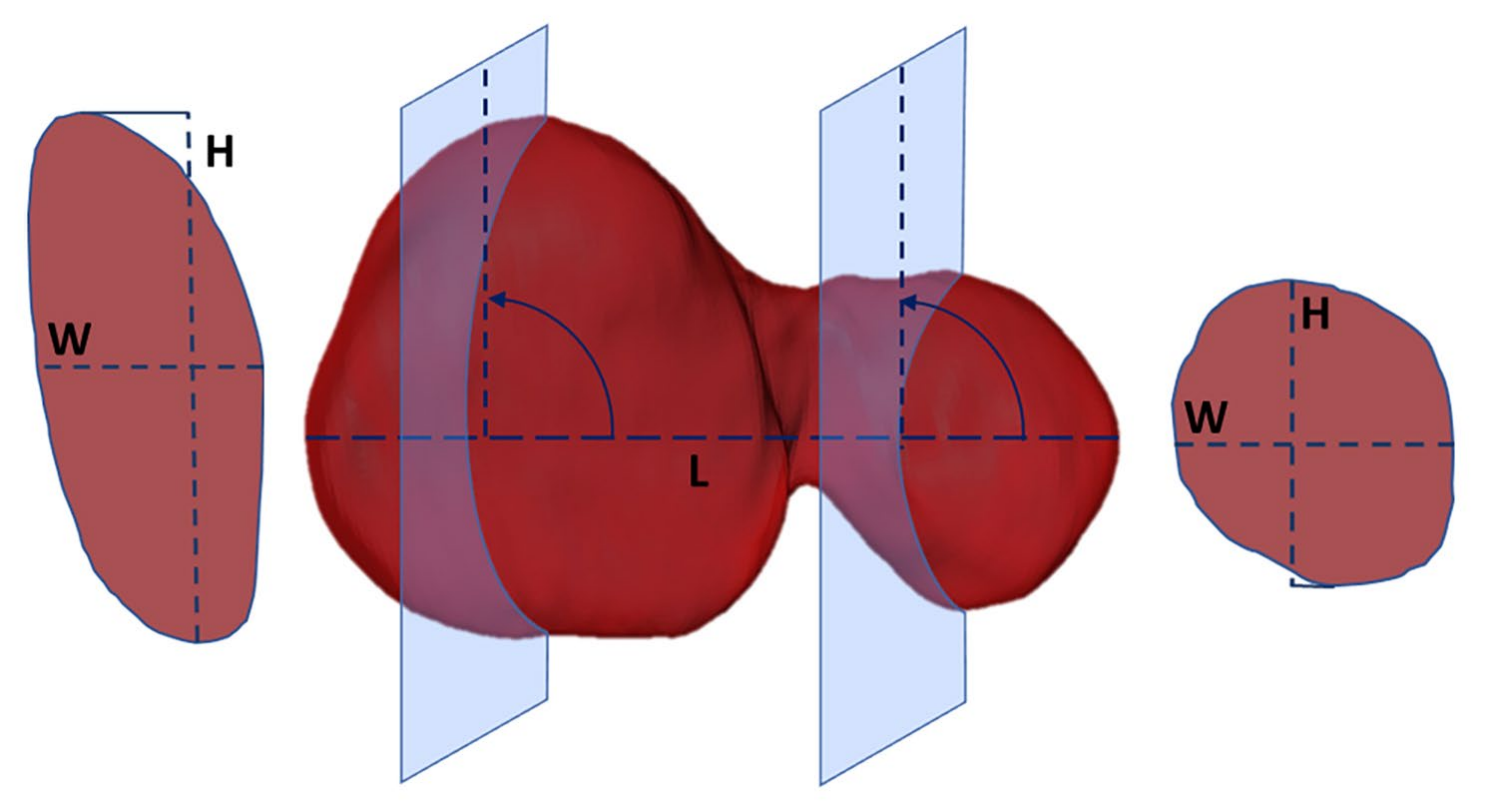
Concept of linear measurements on the adrenal glands. Linear measurements in adjusted CT image plane exemplified for the cranial and caudal poles of the left adrenal gland. Here, the length (L) serves as a vertical line to which the transverse plane is orthogonally aligned. In the adjusted transverse view, height (H) and width (W) were measured as in the illustration

In addition to the volume measurements, the same observer conducted length measurements on each adrenal gland. The surface of the adrenal glands generated from the volume measurement was displayed by the software Amira 6.5 on the respective CT image plane as outer boundary line. Using the three-dimensional length measuring tool in Amira, the maximum length (craniocaudal dimension) was fixed according to the surface boundaries of the adrenal gland in the sagittal CT image plane. The maximal craniocaudal length of the adrenal gland served as a perpendicular line. The transversal CT image plane was adjusted orthogonally to this line in order to provide a correction of individual orientation differences between the adrenal glands by a uniform, standardized sectional view (Fig. 2). On this adjusted transversal CT image plane, maximal height (H (dorsoventral diameter)) and width (W (mediolateral diameter)) were determined for both adrenal glands in the cranial and caudal adrenal pole. The height and width of the isthmus of the left adrenal gland were obtained in the same procedure. Additionally, the dorsoventral aortic diameter caudal to the cranial mesenteric artery was measured in the transversal image plane. To relate the adrenal volume to body size, a ratio between the cubic root of the volume of the respective adrenal gland and the dorsoventral aortic diameter was calculated. This ratio is hereafter abbreviated as “ratio volume-aorta” (RVA).

$$RVA = \left[ {\frac{{\sqrt[3]{{Volume\,of\,adrenal\,gland}}}}{{Aortic\,dorsoventral\,diameter}}} \right]$$

### Statistical analysis

Statistical analysis was carried out using SAS® Enterprise Guide® 7.1 (Statistical Analysis Software, HMS Analytical Software GmbH, Heidelberg, Germany). Normal distributions were evaluated with Shapiro-Wilk test. In case of normal distribution, differences in the mean values of the groups were tested with one-way-analyses of variance (ANOVA) and Ryan-Einot-Gabriel-Welsch multiple range tests (REGWQ). In the absence of normal distribution, Kruskal-Wallis tests and Wilcoxon’s two-sample tests were performed. In the case of the attenuation values, means and standard deviations were also listed in addition to the median and quartiles for the purpose of comparability with other studies. For statistical evaluation of side effects, the paired-sample t-test or the Wilcoxon’s signed rank test was used, depending on the presence of normal distribution. P-values of less than 0.05 defined as statistically significant. Furthermore, relationships between body weight and the respective volume or length measurements of the adrenal glands were determined by the Pearson correlation coefficient. The correlation was classified according to its value as very strong (0.8–1), strong (0.6–0.79), moderate (0.4–0.59), weak (0.2–0.39), and very weak (0.0–0.19). To provide reference values, a 10th and 90th percentile were applied on the present data. The following formula was used to calculate coefficients of variation (CV) of the breed mean values to the different length measurements.

$$C{V_{length}} = \left[ {\frac{{Standard\,deviation\,(SD)\,of\,breed\,mean\,values}}{{Average\,of\,breed\,mean\,values}}} \right]$$

## Results

### Volumetric measurements

Adrenal gland volumes were found to range from 0.34 to 1.93 cm^3^ for the right and from 0.39 to 2.23 cm^3^ for the left side. The mean volume values and standard deviation of the surveyed dog breeds are listed in Table 2. The ANOVA showed significant differences between the dog breeds for the volumetric measurements of the right (F = 24.64; *p* < 0.0001) and left (F = 18.82; *p* < 0.0001) adrenal gland. For both adrenal glands, the Ryan-Einot-Gabriel-Welsch multiple range test (REGWQ) revealed statistically significant differences in adrenal volume between the large (German shepherd, Labrador, Boxer) and smaller (Beagle, Jack Russell Terrier, Dachshund) dog breeds (Table 2). Regarding the volume of the left and right adrenal gland of a breed, no statistically significant side differences were detected in the paired-sample t-test. The Pearson correlation test showed significant positive correlations between the body weight and the volume of the right (r = 0.84; *p* < 0.0001) and left (r = 0.80; *p* < 0.0001) adrenal gland. The aortic diameter used for the RVA revealed a statistically significant positive correlation with body weight (r = 0.82 *p* < 0.0001). There was neither a statistically significant difference between male and female nor between intact and neutered dogs with regard to the RVA of the respective adrenal gland in the Kruskal-Wallis test (*p* > 0.4). Furthermore, based on RVA, significant differences were found between weight groups greater and less than 20 kg body weight for both adrenal glands (left adrenal gland: *p* < 0.0001; right adrenal gland: *p* < 0.0006) using the Wilcoxon two sample test. Thereby, dogs of the smaller weight class showed larger RVA values (Table 3). No significant differences for the RVA were found between breeds of the same weight class. Thereby, for RVA, a 90th percentile of 1.4 for dogs smaller than 20 kg and of 1.1 for dogs of more than 20 kg body weight was determined for the left adrenal gland as an upper reference value. Equivalent upper reference values could be obtained by 90th percentile for the right adrenal gland measuring 1.5 for the lighter weight group and 1.2 for the heavier weight group (Table 3).

**Table 2:** Ryan-Einot-Gabriel-Welsch and Quiot (REGWQ) multiple range test and paired-sample t-test of left and right adrenal volume

Breed group	Number (N)	Mean left adrenal volume (cm³) ± SD	REGWQ Grouping	Mean right adrenal volume (cm³) ± SD	REGWQ Grouping	P-values paired-sample t-test
German Shepherd	10	1.37 ± 0.41	A	1.54 ± 0.29	A	> 0.06
Labrador Retriever	16	1.26 ± 0.31	A	1.34 ± 0.37	A	> 0.24
Boxer	8	1.42 ± 0.29	A	1.44 ± 0.24	A	> 0.82
Beagle	14	0.74 ± 0.13	B	0.76 ± 0.16	B	> 0.43
Jack Russel Terrier	12	0.66 ± 0.19	B	0.67 ± 0.19	B	> 0.60
Dachshund	6	0.71 ± 0.17	B	0.74 ± 0.18	B	> 0.33
Bertolini et al., 2006	48	0.60 ± 0.17		0.55 ± 0.19		

The present table shows the mean volumes of the left and right adrenal glands along with the related standard deviation (SD). Results of REGWQ and paired-sample t-test for the volume of the left and right adrenal gland are shown. Means with the same letter were not significantly different. The *p*-values for the paired-sample t-test lower than 0.05 were representative for significant differences between the sides. N = number of patients

**Table 3:** Percentile for adrenal size-related measurements in the weight groups under and above 20 kg

	Number (N)		Volume (in cm³)		RVA		Height caudal adrenal pole (in mm)	
Dogs of			left	right	left	right	left	right
< 20 kg	32	90th percentile	0.82	0.92	1.44	1.51	8.09	7.06
		**median**	**0.74**	**0.74**	**1.25**	**1.21**	**7.38**	**6.07**
		10th percentile	0.47	0.52	0.93	0.95	6.37	4.88
> 20 kg	34	90th percentile	1.68	1.82	1.14	1.22	9.73	8.52
		**median**	**1.41**	**1.44**	**0.98**	**0.99**	**8.62**	**7.27**
		10th percentile	0.89	0.97	0.83	0.83	7.26	6.04

Weight-related data on volume, the RVA and the height in the caudal adrenal pole of the respective right and left adrenal glands using a median and its quartiles are expressed in the present table

### Length measurements

The 10th and 90th percentiles for height in the caudal adrenal pole for the weight groups are listed in Table 3. Body weight showed a significant positive correlation with the craniocaudal dimension of the right (r = 0.76; *p* < 0.0001) and left adrenal gland (r = 0.81; *p* < 0.0001). The height in the cranial adrenal pole of the right (r = 0.67; *p* < 0.0001) and left side (r = 0.67; *p* < 0.0001) also revealed a significant positive correlation with body weight. Moderate correlations were determined for height (right gland: r = 0.52; *p* < 0.0001, left gland: r = 0.57; *p* < 0.0001) and width (right gland: r = 0.47; *p* < 0.0001, left gland: r = 0.40; *p* < 0.0001) in the caudal adrenal poles and for the height of the isthmus in the left adrenal gland (r = 0.59; *p* < 0.0001) to body weight. Weak correlation was observed for the width in the cranial pole of the left adrenal gland (Table 4).

**Table 4:** Linear adrenal size parameters and associated coefficients of variation

	German Shepherd	Labrador Retriever	Boxer	Beagle	Jack Russel Terrier	Dachshund	CV	Correlation with body weight
Length (L)	30.2 ± 2.9	27.8 ± 2.4	32.2 ± 3.5	22.4 ± 2.4	20.5 ± 2.6	20.3 ± 2.0	0.20	0.81
Length (R)	34.9 ± 3.3	29.2 ± 3.2	31.2 ± 3.7	25.7 ± 3.1	19.1 ± 2.7	21.4 ± 3.6	0.22	0.76
Cranial height (L)	13.2 ± 1.9	12.9 ± 2.2	12.3 ± 1.3	11.0 ± 1.5	10.1 ± 1.2	9.8 ± 1.2	0.13	0.67
Cranial height (R)	17.8 ± 3.0	14.9 ± 2.7	15.7 ± 2.5	12.4 ± 1.1	12.6 ± 2.0	12.7 ± 2.1	0.15	0.67
Cranial width (L)	6.0 ± 0.8	6.4 ± 1.1	6.1 ± 0.6	5.5 ± 1.4	5.9 ± 1.1	6.7 ± 1.2	0.07	NS
Cranial width (R)	6.4 ± 0.6	6.5 ± 1.6	6.2 ± 1.1	5.2 ± 0.9	5.8 ± 1.1	5.9 ± 0.6	0.08	0.35
Caudal height (L)	8.5 ± 1.2	8.3 ± 0.8	8.9 ± 1.4	7.4 ± 0.8	7.0 ± 0.6	7.7 ± 0.9	0.09	0.57
Caudal height (R)	7.2 ± 0.8	7.3 ± 0.9	7.6 ± 1.4	5.8 ± 0.8	6.2 ± 0.8	6.4 ± 0.5	0.11	0.52
Caudal width (L)	7.0 ± 1.2	7.7 ± 1.1	7.1 ± 1.2	6.3 ± 0.8	6.5 ± 1.0	6.8 ± 0.8	0.07	0.40
Caudal width (R)	5.7 ± 0.5	6.7 ± 1.1	6.7 ± 0.8	5.2 ± 0.8	5.5 ± 0.6	5.5 ± 0.9	0.10	0.47
Isthmus height (L)	5.9 ± 0.6	5.5 ± 0.7	6.1 ± 0.8	5.2 ± 0.7	4.4 ± 0.7	4.9 ± 1.0	0.11	0.59
Isthmus width (L)	4.9 ± 1.1	4.9 ± 0.8	4.8 ± 1.0	4.6 ± 0.6	5.0 ± 1.4	5.1 ± 0.9	0.04	NS

Mean values and standard deviation of linear measurements (in mm) are shown in this table. In addition, breed-related coefficients of variation (CV) and Pearson correlations for the respective measurement with body weight are represented in the chart. NS = not significant, L = left adrenal gland R = right adrenal gland

**Fig. 3:**
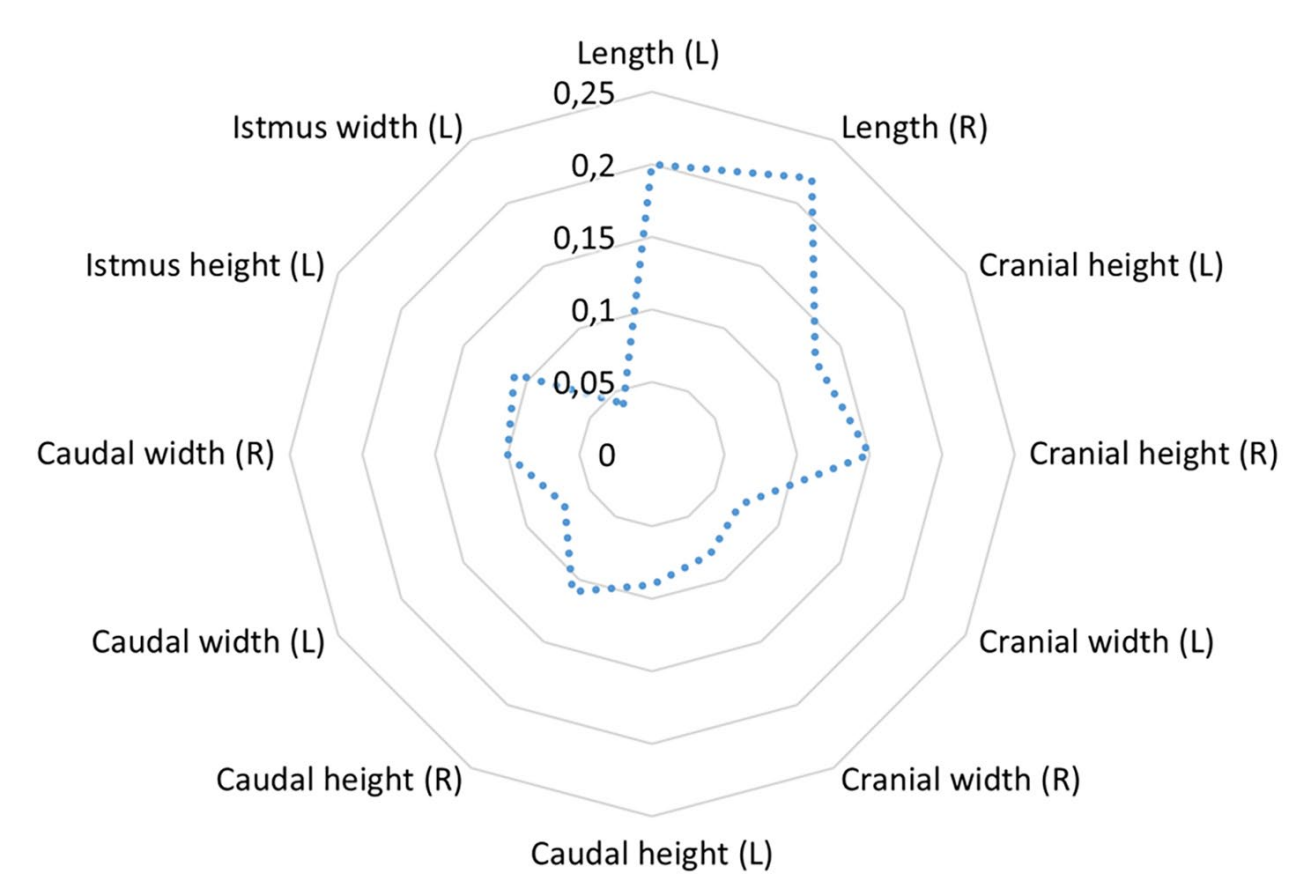
Coefficient of variation for linear measurements on the adrenal glands The coefficient of variation of the respective linear measurements used for comparability and description of breed-related variations in adrenal diameters and length measurements. L = left adrenal gland; R = right adrenal gland

The highest coefficient of variation was obtained for the craniocaudal dimension of the right adrenal gland (CV = 0.22) (Fig. 3), followed by the same length variable of the left adrenal gland (CV = 0.20). Thus, the length ranged from 13.37 to 38.71 mm for the right and from 16.04 to 39.27 mm for the left adrenal gland. The respective width of the cranial and caudal adrenal poles showed lower coefficients of variation relative to the associated heights (Table 4). Except for the width of the right adrenal gland, measurements of height and width in the caudal adrenal pole indicated lower coefficients of variation than their counterparts in the cranial pole. The lowest coefficient of variation was observed for the width of the isthmus (CV = 0.04), which was lower than for the associated height (CV = 0.11).

### Attenuation

In case of attenuation, the Kruskal-Wallis test showed no statistically significant differences between the breeds for the right adrenal gland (*p* = 0.079). However, significant differences between the breeds could be demonstrated for the contralateral side (*p* = 0.003). Thereby, the attenuation of the left adrenal gland in Labrador Retrievers differed significantly from the attenuation in Beagles (*p* = 0.029), Jack Russell Terriers (*p* = 0.001), Dachshunds (*p* = 0.014) and German Shepherds (*p* = 0.037) in the Wilcoxon’s two-sample test. Even the group of Boxers was significantly different to the Jack Russell Terriers (*p* = 0.001) and Dachshunds (*p* = 0.024) regarding the attenuation of the left adrenal gland. Except for Jack Russell Terriers (*p* = 0.31), statistically significant differences in adrenal attenuation were found between the right and left adrenal gland for all breeds in the Wilcoxon’s signed rank test, with higher values for the right adrenal gland (Table 5). The median and quartiles of the studied dog breeds are presented in Table 5.

**Table 5:** Adrenal attenuation measurements in contrast enhanced CT scan; Wilcoxon’s signed rank test in terms of side differences

		German Shepherd	Labrador Retriever	Boxer	Beagle	Jack Russel Terrier	Dachshund
Postcontrast attenuation of the left adrenal gland (HU)	80.1 ± 14.7		68.1 ± 14.1	68.1 ± 10.8	78.8 ± 15.4	89.7 ± 18.9	82.3 ± 9.1
Postcontrast attenuation of the right adrenal gland (HU)	87.7 ± 13.0		78.9 ± 15.4	76.4 ± 9.9	89.4 ± 17.3	92.9 ± 21.6	91.1 ± 13.1
Median of postcontrast attenuation of the left adrenal gland (HU)	83.4		65.0	67.9	79.0	83.1	81.1
Lower quartil	63.3		59.8	62.7	69.5	77.2	73.8
Upper quartil	88.7		73.6	74.9	86.7	98.1	90.6
Median of postcontrast attenuation of the right adrenal gland (HU)	89.9		75.8	76.3	86.5	86.8	86.3
Lower quartil	78.5		68.0	69.9	80.5	75.2	81.7
Upper quartil	96.1		86.6	81.8	91.3	107.5	105.0
Wilcoxon’s signed rank test; P-values	0.002		< 0.0001	0.0078	0.0004	0.31	0.031

In the present table, the postcontrast determined attenuations of the right and left adrenal glands are presented for comparability with other studies on the one hand as mean with standard deviation and further as median with its quartiles. Wilcoxon’s signed rank test for investigation of side differences in terms of attenuation was carried out. The *p*-values below a significance level of 0.05 are considered significantly different from each other

## Discussion

Adrenal pathologies may affect all dogs, with risk factors such as age, breed-related body weight and breed affiliation characterized previously [23]. The dog breeds selected in the present study, representing different weight classes, are described in the literature as predominantly affected with canine hyperadrenocorticism [20, 23–28]. Therefore, the aim of the present study was to obtain size-related information on the unaltered adrenal gland of these clinically relevant breeds. The use of cross-sectional and volume measurements has been described in previous studies as a diagnostic tool for assessing adrenal-associated diseases [2, 3, 11–13, 18]. Thereby, volume measurement by SAT procedures provides an accurate method for assessing the size of various organs [14, 15, 29]. Although a positive correlation between volume and linear measurements of the human adrenal gland has been described, volumetric quantification still showed superior reproducibility in terms of interobserver reliability compared to simple linear measurements [16]. Nevertheless, no data are available on the volumetric size of the unaltered canine adrenal gland considering different weight classes or breeds. Therefore, the aim of the current study was to describe the possible effect of body weight on adrenal size with volume measurements and to reevaluate its influence on common length measurements.

According to the results of the present study, statistically significant differences were observed between the breeds in terms of their adrenal volume. Thus, the large-sized dog breeds showed adrenal volumes approximately twice as large compared to the small-sized breeds regarding the mean values. The strong positive correlations between body weight and adrenal volume indicate significant differences between the breeds. Due to the lack of significant differences observed between dog breeds in the same weight class, an influence of body weight on adrenal volume may be assumed. In contrast to the present results, a previous study of volumetric computed tomographic measurements demonstrated no effect of body weight on adrenal gland volumes [8]. However, in the study by Bertolini et al. (2006) small dog breeds were overrepresented, so that a possible influence of larger dog breeds with higher weight on the adrenal gland volume could not be shown. Some sonographic studies have demonstrated a body weight effect for length measurements of the adrenal gland, which supports the findings of the present study [9, 19, 20].

In the study by Agut et al. (2020), significant differences were found with respect to a measured ratio of adrenal height to aortic diameter with decreasing values for heavier canine populations [30], consistent with the present results of the RVA for adrenal volume. No significant differences were observed in our study between dog breeds in the same weight class. Thus, RVA might be suitable as a method for describing and assessing unaltered adrenal volumetric size in relation to body weight for different breeds. An upper RVA reference value corresponding to a 90th percentile for dogs under 20 kg body weight for the unaltered left adrenal gland of 1.4 and for the right adrenal gland of 1.5 are recommended according to our results. For dogs over 20 kg body weight, however, an upper RVA value of 1.1 for the left adrenal gland and 1.2 for the right adrenal gland must be assumed. Further studies are needed to investigate the applicability of this measurement to adrenal enlargement for altered canine adrenal glands.

In the present study, no sex dimorphism was observed for RVA in either the right or left canine adrenal gland. Furthermore, there was no significant difference between neutered and intact dogs regarding RVA. One possible explanation for this observation could be that unlike in human physiology, the synthesis of the steroid hormone dehydroepiandrosterone (DHEA) in dogs occurs primarily through the gonads [31, 32]. Similar to humans, significant differences in DHEA plasma concentrations have been described between male and female dogs [32]. Unlike in dogs, the primary site of synthesis of the DHEA hormone for humans is localized in the adrenal cortex [33], with sex-related size differences observed for the human adrenal gland [16]. Consistent with the reported inferior role of the adrenal gland in canine DHEA synthesis, no significant sex differences were considered regarding RVA. Sonographic studies failed to detect significant differences for the lengths and cross-sectional measurements used between female and male dogs [20, 34], indicating no volumetric differences between the sexes. This result is also confirmed by a study performed on the ratio of adrenal height to aortic diameter, which was not affected by sex [30].

In terms of length measurements between the selected breeds, the craniocaudal dimension of the right and left adrenal gland showed the highest coefficient of variation. Accordingly, strong correlation between the measured length and body weight was observed. Considerable differences between adrenal length measurements of the normal adrenal gland were reported, supporting our findings regarding widely varying adrenal lengths [10, 21, 35]. Thereby, also different ultrasonographic studies demonstrated a significant effect of body weight on adrenal length [9, 19, 21, 36]. According to the calculated coefficient of variation, the heights in the adrenal poles indicate wider standard deviation relative to the associated width. Height measurements revealed strong correlation in the cranial pole and moderate correlations in the caudal pole with body weight. Consistent with our findings in the case of the height in the caudal adrenal pole, weight-based quantities for cross-sectional measurement were obtained [9, 20, 35]. Nevertheless, regardless of the body weight or breed, an upper threshold of 7.4 mm for the maximal left adrenal diameter was commonly utilized to detect ACTH-dependent hyperadrenocorticism by ultrasonography [21]. However, the mean values for height in the caudal pole of the left adrenal glands were substantially higher than the reported value of 7.4 mm within dogs in the large weight classes, thus leading to a misdiagnosis of ACTH-dependent hyperadrenocorticism. Considering a 90th percentile, an upper reference value for the height in the caudal pole of the unaltered left adrenal gland of 9.7 mm must be adopted for heavyweight dog breeds weighing over 20 kg. In contrast, however, lower values for the height in the caudal adrenal pole were observed for dogs in smaller weight classes, close to the reported threshold, implying a weight dependence. The width showed the lowest coefficient of variation compared to the associated heights, especially in the isthmus, and if present, there was a poor moderate or weak correlation with body weight. Therefore, the volume differences between weight classes might be assumed to be caused by an increase in length and height. In accordance with the present results, the clinical use of weight-unrelated reference for cross-sectional and volume measurements to assess adrenal size must be considered as inappropriate.

The observed attenuation values in our breed groups were equal or smaller in mean values with a higher standard deviation to those reported in a previous study [8]. This may be because Bertolini et al. (2006) used a two-dimensional ROI without including the boundary areas but involving enhanced vascular structures that result in increased attenuation values. Furthermore, there is no information by Bertolini et al. (2006) on the acquisition time of the post-contrast CT scan, which has a considerable impact on the measured attenuation. A previous study demonstrated that the dose of contrast agent and the timing of postinjection CT scanning were the main determinants of peak attenuation for adrenal glands within healthy dogs [37]. Accordingly, the use of a constant injection protocol with discharge of 700 mg iodine/kg over 20 s and a CT scan obtained 30 s after the onset of contrast injection should provide the maximum attenuation values for the adrenal gland [37]. Following the applied standard protocol for abdominal CT scans in the current study, CT scans were performed 49 s after initiating contrast administration. Thus, the measured adrenal attenuation values do not correspond to the maximum peak values reported by Blaser et al. (2016), instead representing the attenuation values temporally posterior to the peak [37]. As comparatively lower contrast agent doses were applied in the present study compared to other studies [8, 37], attenuation values that are lower may be explained according to the reported dose effect by Blaser et al. (2016).

The observed side differences in terms of attenuation could not be confirmed in other studies [8, 37]. In contrast to other study designs, the present study utilized a three-dimensional ROI that included the border regions of the adrenal gland. Thereby, a positional relationship with various anatomical structures has been reported for the right adrenal gland, with contact between the adrenal gland and liver parenchyma as well as close association to the dorsal wall of the vena cava [38, 39]. The attenuation values are mean values determined by measuring the density of each voxel within the ROI. The inclusion of boundary areas in the attenuation measurement might lead to increased attenuation values for the right adrenal gland due to the more difficult demarcation against attached anatomical structures and a resulting partial volume effect [10, 38, 39]. The use of contrast agent to identify and differentiate the adrenal glands from surrounding tissues was judged to be beneficial [11]. Precise segmentation minimizes potential errors in volume measurement and consequently for the selected ROI in terms of the calculated attenuation values in relation to the neighboring structures.

Significant differences in left adrenal attenuation values were observed between breeds, with lower levels in large dog breeds except for German Shepherds. These differences may reflect the difficulties relating to contrast administration for dogs of different weights and breeds. Thereby, a study demonstrated that the variation in scatter of the time to aortic and hepatic peak enhancement was wider in a fixed speed rate injection group compared to a fixed injection duration group [40]. Furthermore, the study showed evidence that a shorter injection duration leads to peak attenuation in a shorter time, while a prolonged injection contributes to a delayed peak attenuation [40]. As the protocol performed in this study routinely used a fixed injection rate of 3 mL/sec, which led to different injection durations between dogs, differences between breeds in adrenal attenuation values may be explicable. To achieve more comparable adrenal attenuations between dog breeds, an individual adjustment regarding start time of the scan and injection rate depending on body weight is suggested for future studies. For such a modification, further data from dynamic CT scans of adrenal glands are needed. Nevertheless, the three-dimensional measured attenuation values reflect the range for adrenal glands of healthy dogs from selected breeds considering a standardized protocol with fixed infusion rate.

Heterogeneous contrast enhancement patterns have been described in the literature for neoplastic-structural changes of the canine adrenal gland, which would be due to the biological behavior (e.g., hemorrhages, infarction)[13]. CT characteristics of post-contrast measured attenuation in the early phase for the described tumor types vary widely in dogs [13, 41] and overlap with our results for the unaltered adrenal glands. Nevertheless, post-contrast attenuation of unaltered adrenal glands may provide the basis to characterize hypoattenuating, heterogeneous structural changes of affected adrenal glands using a three-dimensional ROI.

The major limitation of the present study concerned the lack of validation to the method of SAT by necropsy findings for the adrenal glands. However, in a previous study, the applied SAT was tested for canine prostates, demonstrating a significant relation between the actual prostate volume measured by water displacement and the computed volume with low bias [15]. Although we used the same system, the lack of organ-specific validation could still be a potential source of bias in the volumetric analysis. Therefore, to exclude possible sources of bias and support versatile applicability of volumetric analysis, further studies should continue to focus on an organ-specific validation of the SAT method.

## Conclusion

The present study was able to demonstrate a positive influence of body weight on the volume as well as on the length and height of the adrenal glands in selected dog breeds. In this context, our study confirms the need for weight-associated reference values in relation to the assessment of this endocrine gland. Weight-unrelated reference values regarding an ACTH-dependent hyperadrenocorticism might lead to misdiagnosis. RVA provides a breed-unrelated description of adrenal volumes for weight classes, although its utility in altered adrenal glands needs to be investigated in further studies. Sexual dimorphism and differences regarding castration status were not detected. The present study demonstrates the importance of breed-related data for clinical evaluation of canine adrenal glands with respect to attenuation measurements in enhanced CT scans using a fixed injection rate. Attenuation and volume measurements of the canine adrenal gland in CT images provide information about the adrenal structure, which may help to diagnose neoplasia.

## Data Availability

All data generated or analyzed during this study are included in this published article.

## Abbreviations

CT: Computed tomography
CV: Coefficient of variation
DHEA: Dehydroepiandosterone
H: Height
HU: Hounsfield Units
N: Number
REGWQ: Ryan-Einot-Gabriel-Welsch and Quiot multiple range-test
ROI: Region of interest
RVA: Ratio volume-aorta
SAT: Slice addition technique
SD: Standard deviation
W: Width
